# Is small for gestational age status independently correlated with body composition during childhood?

**DOI:** 10.1007/s00431-022-04723-1

**Published:** 2022-11-29

**Authors:** Foteini Balomenou, Dimitrios Rallis, Filippos Evangelou, Anna Zisi, Kalliopi Balomenou, Nikolaos Tsekas, Meropi Tzoufi, Ekaterini Siomou, Vasileios Giapros

**Affiliations:** 1 Neonatal Intensive Care Unit, School of Medicine, University of Ioannina, Ioannina, Greece; 2 Department of Paediatrics, School of Medicine, University of Ioannina, Ioannina, Greece

**Keywords:** Adiposity, Body composition, Deviant birth weight, Metabolic syndrome, Obesity

## Abstract

This study aims to examine if small for gestation age (SGA) status is correlated with alterations in body composition at prepuberty, independently of other factors, comparing SGA-born children with appropriate for gestational age (AGA)-born children. We examined anthropometrics, waist circumference, body mass index (BMI), six skinfold thickness, and body composition using the method of bioelectrical impedance in 636 children aged 7 to 10 years. We also considered age, gender, birth mode, mother’s age, prepregnancy weight, weight gain during pregnancy, social status, parental BMI, type of feeding, and daily exercise. We examined 636 children at a mean age of 9 years: 106 SGA-born and 530 AGA-born children. SGA as compared to AGA-born children had a lower BMI z-score (0.26 ± 0.89 kg/cm^2^ vs 0.46 ± 0.84 kg/cm^2^, *p* < 0.050) and a lower lean mass, although that was not statistically significant (24.0 ± 6.6 kg vs 25.6 ± 6.4 kg, *p* < 0.100). SGA-born children presented no difference in waist circumference or fat mass in comparison to children born AGA. Logistic regression analysis revealed a strong independent negative association between SGA status and BMI (beta =  − 2.33, OR = 0.70 *p* = 0.019) and SGA status and lean mass (beta =  − 2.43, OR = 0.95 *p* = 0.010).

*  Conclusion*: Our findings suggest that SGA-born children had a lower BMI as compared to AGA-born subjects, whereas SGA status was negatively associated with BMI and lean mass.

**Table:** 

**What is Known:** *• Deviant birth weight for gestation has been associated with an increased risk of childhood adiposity.* *• Evidence remains scarce on whether small for gestational age status affects body composition and obesity later in childhood.*	
**What is New:** *• Among school-aged children, small for gestational age subjects had a lower body mass index as compared to appropriate for gestational age counterparts, whereas small for gestational age status was negatively associated with body mass index and lean mass.* *• A meticulous observation is needed during childhood in children born with deviant birth weight.*

**What is Known:**

*• Deviant birth weight for gestation has been associated with an increased risk of childhood adiposity.*

*• Evidence remains scarce on whether small for gestational age status affects body composition and obesity later in childhood.*

**What is New:**

*• Among school-aged children, small for gestational age subjects had a lower body mass index as compared to appropriate for gestational age counterparts, whereas small for gestational age status was negatively associated with body mass index and lean mass.*

*• A meticulous observation is needed during childhood in children born with deviant birth weight.*

## Introduction 

Childhood obesity has emerged as a global public health problem, associated with certain morbidities including type two diabetes mellitus, hypertension, coronary artery disease, stroke, nonalcoholic liver disease, obstructive sleep apnea, dyslipidemia, and several types of cancer, as well as severe psychological consequences [1]. Several genetic, environmental, and biological factors have been implicated in the pathophysiology of excess weight gain during childhood [2–4]. Among them, birth weight has been identified as a significant indicator of the in utero experiences of the fetus that may have an impact on later life [3].

The term “small for gestational age (SGA)” is used to define an infant whose birth weight is less than the 10th percentile for gestational age or two standard deviations below the population norms on the growth charts, not considering the intrauterine growth trajectory [5]. The term “intrauterine growth restriction (IUGR)” refers to the condition characterized by a fetal growth deceleration and/or clinical features of intrauterine malnourishment, irrespective of the birth weight percentile in relation to gestational age [5]. IUGR fetuses represent those fetuses that have failed to reach their growth potential, due to genetic, infectious, maternal, or placental disorders [6]. Current evidence suggests that SGA and IUGR neonates are at an increased risk of developing metabolic complications in later life [5].

Previous studies have identified that SGA status is a significant factor associated with decreased body mass index (BMI) or fat mass during childhood [7–9], whereas, on the other hand, several others have suggested that SGA-born subjects present an increased risk of later fat accumulation and childhood obesity [10–13]. As previously suggested, fetal growth restriction and adiposity accumulation in later life share environmental and genetic antecedents [14]. It has been assumed that growth-retarded fetuses develop a remodeling of their body composition in utero, whereas they show faster postnatal growth [14], and they experience a significant adiposity accumulation when exposed to a nutrient-rich environment [15]. On the other hand, recent evidence has suggested that numerous other factors, irrespective of birth weight, are important in determining later fat mass and body composition [16]. Given the conflicting results arising from different studies, a debate is still ongoing [14, 16, 17].

This study aimed to examine if SGA status is correlated with alterations in body composition at prepuberty independent of other prenatal, perinatal, and postnatal factors by comparing children born SGA with those born appropriate for gestational age (AGA).

## Material and methods

### Study design and population

This prospective study was conducted from 2019 to 2020 in the city of Ioannina, in Northwestern Greece, including children between 7 to 10 years of age. The local education bureau gave us permission to conduct the study in school, whereas the study was approved by the Science and Research Ethics Committee of the University of Ioannina (No. 934).

A representative sample of schools in the urban area was selected for the initial recruitment of the children. Children and their caregivers were approached in their school settings or were contacted by telephone and asked to participate in the study. After the purpose of the study was explained, written informed consent was obtained from each parent, in addition to oral consent that was obtained from the early school-aged participants.

A total of 710 children were enrolled to participate, and they were further classified based on their birth weight for gestation. Anthropometrics were obtained from the children’s medical records. The AGA group consisted of children with birth weight for gestational age between the 10th and 90th centile, according to the new INTERGROWTH charts [18]. SGA group consisted of children with a birth weight below the tenth centile, whereas the large for gestational age (LGA) consisted of children with a birth weight above the 90th centile, according to the same growth charts.

To avoid a potential bias due to constitutional factors leading to SGA, we excluded SGA-born children whose mothers’ height was below two standard deviations of the mean or whose mothers had a height between one to two standard deviations of the mean and had also given birth to an SGA infant previously [19, 20]. We excluded 24 children due to missing information on the predictor or outcome variables or suspicion of inaccurate measurements and 50 children as they were LGA. Thus, 636 children in total comprised the study population, 106 SGA-born and 530 AGA-born subjects. Based on the annual number of births in the urban area of Ioannina, it was estimated that the study sample represented 22–24% of the children aged 7 to 10 years living in the area.

### Predictor variables

Data on the birth history of every child participating in the study, including gestational age, gender, birth weight, delivery order, mode of delivery, and birth length, was obtained from every individual’s medical record that was provided by his/her caregiver. Children born below 35 weeks were not included in this study, and also, none of the children included in the study had any evidence of congenital infections, genetic syndromes, or congenital malformations. The ponderal index (PI) was calculated according to the formula *PI* = *weight* × *100/length*^*3*^* (g/cm*^*3*^*)*. The children’s caregivers were also asked to fulfill a questionnaire including information regarding pregnancy (i.e., maternal age, weight gain during pregnancy, pregnancy pathology including gestational diabetes, or hypertension), nutritional data (i.e., exclusively breastfeeding, formula feeding, or mixed feeding at birth, breastfeeding at 6 months, duration of breastfeeding, time of solid food introduction), duration of weekly exercise, parental BMI, and family socioeconomic status. The duration of weekly exercise was calculated by the sum of hours that children were dedicated to out-of-school sports activities. The socioeconomic status of the family was classified as high, medium, or low, based on the level of parental education and the annual income.

### Outcome variables

Children were assessed at their school or at the outpatient clinic of the Neonatal Unit of the University Hospital, where we measured weight, height, waist circumference, six skinfold thickness, blood pressure, and body composition. All measurements were performed at least 3 h after the morning awakening and after a minimum of three-hour fasting. Children’s caregivers had been also advised to prevent children from intense exercise within the preceding 12 h before the measurement. Finally, children had their measurements performed after voiding. Weight was measured with an electronic scale (Tanita-DC 430-U, Tanita Corporation, Tokyo, Japan) to the nearest 0.1 kg and height by a Harpenden stadiometer to the nearest 0.5 cm (Seca, Vogel & Halke GmbH & CO. KG, Hamburg, Germany). Waist circumference was measured twice to the nearest 1 mm at the minimum circumference between the iliac crest and the rib cage, expressed as a z-score. The BMI was calculated based on the formula *BMI* = *weight/height*^*2*^* (kg/m*^*2*^*)* and was expressed as a z-score. To calculate the z-score for any BMI value, we used centile charts of the World Health Organization [21]. All anthropometric measurements were performed twice, and the mean value was used for analysis. The sum of the thickness of the skinfolds was assessed (Calipers, UK), measuring the double fold of skin and subcutaneous fat at the triceps (the midpoint of the back of the upper arm), the biceps (at the same level, to the front of the upper left arm), the subscapular (below and laterally to the shoulder blade), the suprailiac obliquely, the midthigh, and the midcalf. The predicted mass density (PMD) was calculated according to the formula *PMD* = *1.1533 − (0.0598* × *L)* for males and *PMD* = *1.1369 − (0.0643* × *L)* for females (*L* represents the logarithm of the cumulative sum of the four-skinfold thickness in mm), whereas the percentage (%) of body fat was calculated from the Siri equation [*% Body Fat* = *(495/PMD) − 450*].

Blood pressure was measured non-invasively in the right arm twice in every child while sitting (HS 20S sphygmomanometer, Shenzhen, China), and the values were assessed according to the updated criterion of the Fourth Report on the Diagnosis, Evaluation, and Treatment of High Blood Pressure in Children and Adolescents of the American Academy of Paediatrics [22].

Body composition was evaluated with Bioelectrical Impedance Analysis (BIA) (Tanita-DC 430-U, Tanita Corporation, Tokyo, Japan), measuring absolute fat mass, fat mass percentage, absolute lean mass, lean mass percentage, absolute total body water, total body water percentage, and total body weight. Lean mass was calculated using the cross-validated equation by Houtkooper [23]. BIA measures the impedance of body tissues to the flow of an alternating electric current, based on the differing electrolyte and water content of tissues and the composition of cell membranes [24, 25].

### Statistical analysis

Descriptive statistics were calculated for collected characteristics. Continuous variables were expressed as mean ± standard deviation, while categorical variables were expressed as *n* (percentage %). The normality of the distributions of continuous variables was assessed by the Kolmogorov–Smirnov test. Comparisons between continuous variables were performed utilizing the Student’s *t* test and between categorical variables with the chi-square (*x*^2^) test or Fisher’s exact test. Logistic regression analysis was used to estimate the association between SGA status (dependent variable) and body composition at prepuberty (either lean mass or fat mass) or BMI after taking into consideration factors that may affect body composition. Namely, we included gestational age, gender, delivery order, mode of delivery, maternal age, weight gain during pregnancy, and pregnancy pathology including gestational diabetes or hypertension, nutritional data, i.e., exclusively breastfeeding, formula feeding, or mixed feeding at birth, breastfeeding at 6 months, duration of breastfeeding, and time of solid food introduction, duration of weekly exercise, parental BMI, and family socioeconomic status.

In sample size analysis, a cohort of more than 500 subjects was powered to detect differences of 10% in fat mass, lean mass, or BMI between the SGA and AGA subjects, with a power of 0.8 and a type-I error of 0.05. All performed tests were two-sided, and a *p* value less than 0.05 was considered statistically significant (alpha 0.05). The data were analyzed using StatView (SAS, CA USA).

## Results

Among 636 children who were examined at a mean age of 9 years, 106 (17%) were SGA and 530 (83%) were AGA. The gestational age (37.0 ± 1.9 weeks), birth weight (2262 ± 355 g), and length at birth (47.1 ± 2.0 cm) of SGA-born subjects were significantly lower in comparison to AGA-born subjects (38.6 ± 1.2 weeks, 50.5 ± 1.0 cm, and 3200 ± 282 g, respectively, *p* < 0.001). SGA as compared to AGA subjects were born predominantly with a cesarean section (72% in comparison to 34%, *p* < 0.001), whereas SGA had an SGA-born sibling in 24%, as compared to 3% of the AGA-born children (*p* < 0.001). No significant differences were noted between the two groups regarding gender, delivery order, pregnancy weight gain, gestational diabetes, or hypertension. Regarding the parental characteristics, we recorded a significantly lower maternal weight and height among mothers of the SGA as compared to AGA children, but no differences in maternal age, maternal BMI, paternal weight, height, or BMI, as depicted in Table 1.

**Table 1:** Perinatal, maternal, and paternal characteristics between SGA and AGA children

	**AGA (*n* = 530)**	**SGA (*n* = 106)**	***p***
Gestational age, weeks	38.6 ± 1.2	37.0 ± 1.9	< 0.001
Birth weight, g	3200 ± 282	2262 ± 355	< 0.001
Birth length, cm	50.5 ± 1.0	47.1 ± 2.0	< 0.001
Mode of delivery, cesarean section	178 (34%)	76 (72%)	< 0.001
Previous SGA birth	15 (3%)	25 (24%)	< 0.001
Sex, male	251 (47%)	49 (46%)	n/s
Delivery order (1/2/ > 2)	326/158/46	74/27/5	n/s
Ponderal index, g/cm^3^	2.49 ± 0.2	2.17 ± 0.2	n/s
Pregnancy weight gain, kg	12.9 ± 6	13.0 ± 6	n/s
Gestational diabetes	40 (8%)	14 (13%)	n/s
Gestational hypertension	13 (2%)	2 (1%)	n/s
Maternal age, years	31 ± 4	31 ± 4	n/s
Maternal height, cm	165 ± 5	164 ± 5	< 0.010
Maternal weight, kg	65 ± 11	62 ± 11	< 0.050
Maternal BMI, kg/m^2^	24 ± 4	23 ± 4	n/s
Paternal height, cm	179 ± 6	179 ± 6	n/s
Paternal weight, kg	86 ± 12	86 ± 14	n/s
Paternal BMI, kg/m^2^	26 ± 3	27 ± 3	n/s

Continuous variables are expressed as mean ± standard deviation. *p* values of Student’s *t* test. Categorical variables are expressed as *n* (%). *p* values of chi-square test or Fisher’s exact test

*SGA* small for gestational age, *AGA* appropriate for gestational age, *BMI* body mass index

SGA in comparison to AGA-born children had a lower ratio of exclusively breastfeeding at birth (30% as compared to 50%, *p* < 0.001), a shorter duration of breastfeeding (5.2 ± 4.5 months in comparison to 7.6 ± 4.3 months, *p* < 0.050), and a lower ratio of breastfeeding at 6 months of age (19% in comparison to 34%, *p* < 0.001). We recorded no differences between the two groups regarding the time of solid food initiation, the duration of weekly exercise, or socioeconomic status (Table 2).

**Table 2:** Characteristics of nutrition, exercise, and the socioeconomic status between SGA and AGA children

	**AGA (*n* = 530)**	**SGA (*n* = 106)**	***p***
Exclusively breastfeeding at birth	263 (50%)	32 (30%)	< 0.001
Exclusively formula feeding at birth	154 (29%)	48 (45%)	< 0.001
Mixed feeding at birth	113 (21%)	26 (25%)	n/s
Breastfeeding duration, months	7.6 ± 4.3	5.2 ± 4.5	< 0.050
Breastfeeding at 6 months	178 (34%)	20 (19%)	< 0.001
Solid food initiation, months	5.9 ± 0.6	6.1 ± 0.5	n/s
Duration of weekly exercise, hours	3.7 ± 2.4	3.6 ± 3.3	n/s
Socioeconomic status (high/medium/low)	53/190/287	7/44/55	n/s

Continuous variables are expressed as mean ± standard deviation. *p* values of Student’s *t* test. Categorical variables are expressed as *n* (%). *p* values of chi-square test or Fisher’s exact test

*SGA* small for gestational age, *AGA* appropriate for gestational age

The mean age of assessment was 9.1 ± 1.6 years for SGA-born children and 9.2 ± 1.6 years for AGA-born children. SGA had a significantly lower BMI z-score (0.26 ± 0.9 kg/cm^3^ in comparison to 0.46 ± 0.8 kg/cm^3^, *p* < 0.050). We recorded no significant differences between SGA and AGA-born children in the waist circumference z-score, the sum of six skinfold thickness, the percentage of fat mass estimated with the Siri equation, the PMD, the absolute fat mass, or the percentage of fat mass. We found a lower lean mass of 24.0 ± 6.6 kg in SGA-born children in comparison to 25.6 ± 6.4 kg in AGA-born children, although that was not statistically significant (*p* < 0.100). Finally, we recorded significantly lower systolic and diastolic blood pressure in the SGA, as compared to AGA-born subjects (Table 3).

**Table 3:** Anthropometrics, the sum of six skinfold thickness, Siri equation, predicted mass density, body mass composition, and blood pressure in SGA and AGA children at a mean age of 9 years

	**AGA (*n* = 530)**	**SGA (*n* = 106)**	***p***
Age, years	9.1 ± 1.6	9.2 ± 1.6	n/s
Weight, kg	34.2 ± 10	32.6 ± 11	n/s
Height, cm	136.8 ± 12	135.5 ± 12	n/s
BMI z-score, kg/m^2^	0.46 ± 0.8	0.26 ± 0.9	< 0.050
Waist circumference z-score, cm	0.00 ± 0.58	− 0.14 ± 0.56	n/s
Sum of six skinfold thickness, cm	33.6 ± 16	32.6 ± 18	n/s
Biceps, cm	5.6 ± 3.0	5.2 ± 3.1	n/s
Triceps, cm	5.2 ± 2.9	4.9 ± 2.9	n/s
Subscapular, cm	5.7 ± 2.5	5.5 ± 2.4	n/s
Suprailiac, cm	8.7 ± 4.3	8.7 ± 4.6	n/s
Midthigh, cm	3.1 ± 2.9	3.0 ± 3.1	n/s
Midcalf, cm	5.2 ± 3.0	5.2 ± 3.2	n/s
Siri equation (% fat mass)	18.6 ± 6.6	18.7 ± 6.9	n/s
Predicted mass density, g/ml	1.06 ± 0.15	1.06 ± 0.16	n/s
Fat mass, kg	7.4 ± 4.3	7.2 ± 4.9	n/s
Fat mass, percentage (%)	20.4 ± 6.7	20.1 ± 7.9	n/s
Lean mass, kg	25.6 ± 6.4	24.0 ± 6.6	< 0.100
Systolic blood pressure, mmHg	100 ± 12	98 ± 11	< 0.050
Diastolic blood pressure, mmHg	66 ± 8	64 ± 8	< 0.050

Continuous variables are expressed as mean ± standard deviation

*p* values of Student’s *t* test, *SGA* small for gestational age, *AGA* appropriate for gestational age, *BMI* body mass index

In logistic regression analysis, we found a strong independent negative association between SGA status and lean mass (beta =  − 2.43, OR = 0.95, *p* = 0.010) and SGA status and BMI (beta =  − 2.33, OR = 0.70, *p* = 0.019) (Table 4).

**Table 4:** Logistic regression analysis of the adjusted effect of SGA status on BMI and body composition

	***p***	**Beta (β)**	**Odds ratio (OR)**	**Confidence interval (CI)**
BMI	0.019	− 2.33	0.70	0.55–0.94
Lean mass	0.010	− 2.43	0.95	0.91–0.99
Fat mass	0.160	− 1.30	0.96	0.91–1.01
Fat mass, percentage	0.210	− 1.20	0.98	0.94–1.01
Waist circumference z-score	0.130	− 1.33	1.00	0.74–3.80

*SGA* small for gestational ag, *BMI* body mass index

## Discussion

The findings of the current study suggest that SGA-born children that were evaluated at a mean age of 9 years had a significantly lower BMI, as compared to AGA-born counterparts. SGA status was negatively associated with BMI and lean mass at school age.

A previous study that was performed by Rogers et al. included more than 6000 children and examined their body composition according to their birth anthropometrics with dual-energy x-ray absorptiometry (DeXA) [9]. The authors concluded that birth weight and birth length were associated with body composition and fat distribution during childhood [9]. In particular, birth weight and the ponderal index were both positively associated with the accumulation of fat and lean mass [9]. Our findings are in part in agreement with the above study, suggesting that SGA-born children had a significantly lower BMI and lower lean mass, although that was not statistically significant, as compared to AGA-born subjects. Of note, Rogers et al. examined the association of birth weight with fat mass accumulation during childhood in a linear model rather than categorically with a subgroup analysis between SGA and AGA-born children [9]. Although birth weight is a continuous variable, SGA-born children are traditionally considered a pathophysiological district subgroup.

Hediger et al. examined the association between birth weight and body composition among SGA, AGA, and LGA-born children [7]. The study included 3192 children that were evaluated with skinfold thickness at 3 to 6 years of age [7]. The authors suggested that SGA-born subjects had a trend of reduced lean mass development and that various environmental factors might have influenced fat mass development; however, they found no significant association between birth weight and fat mass accumulation, apart from a short period of the study [7]. The differences in the findings regarding the fat mass accumulation between the previous and our study might be explained by the fact that, in the previous study, children were examined at a much younger age in comparison to our cohort and that the fat mass was solely assessed by skinfold thickness, which, although useful in large epidemiological studies, is less accurate in comparison to BIA of DeXA [7].

Besides, Kramer et al. performed a large pertinent study evaluating 17,046 children at 11.5 years of age with anthropometrics, skinfold thickness, and BIA [8]. The authors reported that children born SGA had indeed a lower BMI and a lower percentage of body fat as compared to AGA-born children [8]. Although the findings of our study were in agreement with the above regarding BMI, we could not find any significant association between body weight deviation and fat mass. Interestingly, the differences in the fat mass that were recorded in the previous study were only 0.5%. Among others, those differences might be explained by the fact that the study was performed in Belarus, a country with a low prevalence of obesity. Furthermore, the authors in the design of their study excluded newborns with a birth weight below 2500 g, which could represent those with the greatest degree of fetal growth restriction [8].

Our findings suggested that SGA subjects had no differences as compared to AGA children in the waist circumference and body fat mass, although we could not evaluate the central fat distribution. SGA as compared to AGA-born subjects presented a lower BMI, whereas lean mass was lower but did not reach statistical significance. A lower BMI in SGA children along with a similar waist circumference may indirectly imply that SGA-born children presented a concentric distribution of the fat. Previous studies have suggested that SGA-born children present an increased fat mass accumulation which is predominantly centrally distributed [10–13]. Crume et al. demonstrated that fetal growth-restricted children tended to present higher subcutaneous adipose tissue at a mean age of 10 years in MRI [10]. Nevertheless, Ibanez et al. in a longitudinal study of 51 children evaluated with DeXA from 2 to 4 years of age reported that SGA-born children had more body fat and abdominal fat mass compared to AGA-born children [11]. Furthermore, the same group evaluated the fat composition with DeXA and MRI in 64 children at 6 years of age and similarly found that SGA children had a viscerotropic redistribution of abdominal fat in comparison to AGA children [13].

Interestingly, in our study, SGA-born children had lower systolic and diastolic blood pressure in comparison to AGA-born subjects. Although we recorded a minimum level of difference (approximately 3%) between the two groups, that was statistically significant. Our findings contradict previous evidence that suggests that SGA-born children are at increased risk of hypertension [26–28]. Previous studies and meta-analyses support that birth weight is inversely associated with the risk of high blood pressure (the “small baby syndrome hypothesis”) [26–28], which can be in part explained by the small size, reduced number, and abnormal development of the nephrons in children with low birth weight [28]. In our study, SGA-born subjects had a significantly lower BMI in comparison to AGA-born counterparts; given that BMI in school age has a significant impact on arterial blood pressure, that might explain the differences in blood pressure noted in our cohort.

Finally, our findings revealed that the rate and duration of breastfeeding were lower in SGA-born in comparison to AGA-born children, suggesting that excessive formula feeding is a common practice in SGA-born infants. This may be partly attributable to the higher pressure that encounter parents of SGA infants seen them to grow rapidly. Breastfeeding has been suggested to have a protective effect against later overweight; however, evidence remains inconsistent since many studies could not find any association between breastfeeding and childhood overweight [29]. In our study, multiple regression analysis could not reveal any significant association between breastfeeding and BMI or lean mass.

The limitations of our study should be acknowledged. Firstly, the study was performed in a single center, including a relatively limited sample population. Although the study population was representative of 22–24% of the children aged 7 to 10 years living in the area, we should be cautious before generalizing our findings. Moreover, we performed the body fat analysis with BIA and the sum of six skinfold thickness, which are indirect methods to evaluate body composition. Air displacement plethysmography, DeXA, and magnetic resonance imaging (MRI) due to their accuracy in body composition evaluation are considered reference methods [30]. Plethysmography uses the principles of body density and is considered a good reference method in serial evaluation to estimate total adiposity, lean mass, and fat mass and can be used in children from 2 years of age. However, body composition can also be estimated by anthropometry and BIA, when population size is extensive, or when economic resources are not available [24, 31].

In conclusion, among school-aged children, SGA had a lower BMI as compared to AGA-born subjects. SGA status was negatively associated with BMI and lean mass. Given that childhood is a crucial period with a major impact on the later development of certain diseases such as obesity or metabolic syndrome, a longitudinal follow-up of SGA-born subjects is warranted beyond infancy and childhood.

## Data Availability

Non-available.

## Abbreviations

AGA: Appropriate for gestational age
BMI: Body mass index
DeXA: Dual-energy x-ray absorptiometry
LGA: Large for gestational age
PI: Ponderal index
PMD: Predicted mass density
SGA: Small for gestational age
